# Supplementary material for: OBINTER: A Holistic Approach to Catalyse the Self-Management of Chronic Obesity
Source: Sensors (Basel). 2020 Sep 6;20(18):5060. doi: 10.3390/s20185060 (PMC7570655; doi:10.3390/s20185060)
Supplement: Supplementary file 1 [file sensors-20-05060-s001.pdf]

Participant information Sheet – Obesity: from molecular biology to intervention (OBINTER)- Usability Evaluation

The Project Team

The OBINTER Consortium, consists of:

- Fundación Biofísica Bizkaia
- Fundación Azti
- Asociación Instituto Investigación Sanitaria Biocruces
- UPV/EHU – Departamento Farmacia y Ciencias de los Alimentos
- Fundación Centro de Tecnologías de Interacción Visual y Comunicaciones - Vicomtech

A team working on this Project, wish to evaluate the current Technology that has been developed within the OBINTER project.

Objectives

Obesity: from molecular biology to intervention (Elkartek programme, Basque Country 2019). This project is based on the hypothesis that by identifying and integrating new key molecular biomarkers of different kinds (metabolomics, lipidomics and membrane biophysics, epigenetics, microbiomics) together with digital 4.0 tools (Big data, algorithms aided by decisions and digital devices), it is possible to define nutritional recommendations and personalized interventions on lifestyles in the general population, with greater efficiency, to promote and improve the prevention programs promoted by the Department of Health and Osakidetza, thereby reducing the incidence of obesity in the Basque Country and associated morbidity and mortality. In short, it will be possible to improve the quality of life of the population and reduce the related health expenditure.

To better understand how well the system is developing we wish to collect demographic data and observe how you interact with the system using screen capture, video, audio record, and ask you to complete a number of questionnaires that will help the team understand how well the system is performing.

We may ask you to participate in other sessions at later dates and compare your interaction at various times during the OBINTER project.

This is an evaluation and should not be considered a research project at this stage.

Screen Capture, Video and Audio recordings

Although the user's interaction with the application on screen, audio and video will be recorded, the access and use of this content will be limited exclusively to members of the OBINTER team for their analysis and usability evaluation (unless it is required by regulatory entities for verification). The recorded content (screen capture and video and audio recording) will be destroyed once it is transcribed and / or its analysis is finished (at the latest at the end of the project).

Any personal identifier collected in the audio will be removed and never transcribed. All information will be securely stored with a pseudo-code and with limited access to the OBINTER team.

If you have any questions about this project or the usability evaluation, please contact Gorka Epelde at Vicomtech gepelde@vicomtech.org .

OBINTER Project

Participant Identification Number:

CONSENT FORM

Title of Project: Usability Evaluation

Please initial box

1. I confirm that I have read the statement of interest dated 6th July 2020 for the above evaluation. I have had the opportunity to consider the information, ask questions and have had these answered satisfactorily ☐

2. I understand that my participation is voluntary and that I am free to withdraw at any time without giving any reason, without my legal rights being affected. ☐

3. I understand that data collected during the project, may be looked at by individuals from the OBINTER project or from regulatory authorities where it is relevant to my taking part in this project. I give permission for these organisations to have access to the data. ☐

4. I understand that the research team will screen-capture, audiotape and potentially videotape me during the evaluation. The information will be held for the duration of the OBINTER project. All of this evaluation data will be destroyed at the end of the OBINTER project. ☐

5. I agree to take part in the above project. ☐

______________________	______________________	______________________	
Name of Participant	Date	Signature	
______________________	______________________	______________________	______________
Name of Person taking consent	Organisation	Date	Signature

Hoja de información del participante – Obesidad: de la biología molecular a la intervención (OBINTER)- Evaluación de Usabilidad

El equipo de proyecto

El consorcio OBINTER se compone de:

- Fundación Biofísica Bizkaia
- Fundación Azti
- Asociación Instituto Investigación Sanitaria Biocruces
- UPV/EHU – Departamento Farmacia y Ciencias de los Alimentos
- Fundación Centro de Tecnologías de Interacción Visual y Comunicaciones - Vicomtech

El equipo de trabajo está trabajando en este proyecto y les gustaría evaluar los desarrollos tecnológicos realizados hasta la fecha en el proyecto OBINTER.

Objetivos

Obesidad: de la biología molecular a la intervención (OBINTER) (Elkartek, Gobierno Vasco 2019). Este proyecto se fundamenta en la hipótesis de que mediante la identificación e integración de nuevos biomarcadores moleculares clave de diferente índole (metabolómica, lipidómica y biofísica de la membrana, epigenética, microbiómica) junto con herramientas digitales 4.0 (Big data, algoritmos a la ayuda de decisiones y dispositivos digitales), es posible definir recomendaciones nutricionales e intervenciones personalizados sobre los estilos de vida en la población general, con una mayor eficiencia, para impulsar y mejorar los programas de prevención promovidos por el Departamento de Salud y Osakidetza, reduciendo con todo ello la incidencia de la obesidad en la CAPV y la morbi- mortalidad asociadas. En definitiva, será posible mejorar la calidad de vida de la población y disminuir el gasto sanitario relacionado.

Puede ser que te volvamos a contactar para contrastar futuras versiones de los aplicativos desarrollados en el proyecto OBINTER.

El este estudio al que se invita mediante este documento se limita a la evaluación de usabilidad y no conlleva la participación en un estudio de investigación clínica.

Captura de pantalla y grabación de video y audio

Aunque se procederá a grabar la interacción del usuario con la aplicación en pantalla , audio y el video, el acceso y uso de este contenido, se limitará exclusivamente a miembros del equipo de OBINTER para su análisis y evaluación de usabilidad (a no ser que sea requerido por las entidades de regulación para su comprobación). El contenido grabado (captura de pantalla y grabación de video y audio), se de destruirá una vez se transcriba y/o termine su análisis (como muy tarde al término del proyecto).

Cualquier identificador personal recogido en el audio será eliminado y nunca transcrito. Toda la información será seguramente guardada con un seudocódigo y con acceso limitado al equipo de OBINTER.

En caso de duda sobre el proyecto o el estudio de usabilidad por favor ponte en contacto con Gorka Epelde del centro tecnológico Vicomtech gepelde@vicomtech.org .

PROYECTO OBINTER

Número identificativo del participante:

FORMULARIO DE CONSENTIMIENTO

Título del estudio: Evaluación de usabilidad Julio 2020

Por favor selecciona las cajas

1. Confirmo haber leído la explicación del estudio con fecha 6 de Julio 2020 para la evaluación indicada arriba. He tenido la oportunidad de evaluar la información, realizar preguntas y recibir respuesta satisfactoria de ellas. ☐

2. Entiendo que mi participación es voluntaria y que estoy en mi derecho de dejar prueba en cualquier momento, sin dar una razón, sin que mis derechos legales se vean afectadas ☐

3. Entiendo que los datos recogidos durante la evaluación puedan ser revisados y analizados por personas que trabajan en el proyecto OBINTER o por las autoridades regulatorias. Doy mi permiso de acceso a dichas organizaciones para acceder a los datos / información provista ☐

4. Entiendo que el equipo de investigación grabará el audio, la pantalla de las aplicaciones testeadas y potencialmente el video de la evaluación. La información se guardará durante la duración de OBINTER. Toda la información de evaluación será eliminada una vez finalice el proyecto. ☐

5. Confirmo mi interés en participar en la evaluación de usabilidad para el proyecto OBINTER. ☐

____________________	____________________	____________________
Nombre del participante	Fecha	Firma

____________________	____________________	____________________	____________________
Nombre de la persona recogiendo el consentimiento	Organización	Fecha	Firma
